# TomoRay cranial: synthesis of cranial CT imaging from biplanar radiographs using a generative adversarial network

**DOI:** 10.1007/s00330-025-12253-1

**Published:** 2026-01-15

**Authors:** Olivier Zanier, Seungjun Ryu, Raffaele Da Mutten, Sven Theiler, Alessandro Carretta, Giorgio Palandri, Diego Mazzatenta, Luca Regli, Carlo Serra, Victor E. Staartjes

**Affiliations:** 1 Machine Intelligence in Clinical Neuroscience & Microsurgical Neuroanatomy (MICN) Laboratory, Department of Neurosurgery, Clinical Neuroscience Center, University Hospital Zurich, University of Zurich, Zurich, Switzerland; 2 Department of Oncology, University of Oxford, Oxford, United Kingdom; 3 Department of Neurosurgery, School of Medicine, Eulji University, Daejeon, South Korea; 4 Institute for Basic Science (IBS), Center for Memory and Glioscience, Daejeon, South Korea; 5 Department of Biomedical and Neuromotor Sciences (DIBINEM), University of Bologna, Bologna, Italy; 6 Programma Neurochirurgia dell’Ipofisi—Pituitary Unit, IRCCS delle Scienze Neurologiche di Bologna, Bologna, Italy; 7 Neurosurgery Unit, IRCCS Instituto delle Scienze Neurologiche di Bologna, Bologna, Italy

**Keywords:** Deep learning, Neuroimaging, Neurosurgery, Tomography (X-ray computed), Head

## Abstract

**Objectives:**

Besides clinical examination, cranial CT plays a critical role in diagnostics in neurosurgery. In trauma cases or perioperatively, having low-barrier access to CT-like imaging would be highly beneficial. Therefore, this feasibility study examines at an early stage if and how well synthetic cranial CT imaging can be generated from biplanar radiographs of adult neurosurgical patients using deep learning.

**Materials and methods:**

Two 2D to 3D generative adversarial networks (GANs) were trained for the generation of synthetic cranial CTs using radiographs taken in two planes as input. Model 1 uses digitally reconstructed radiographs (DRRs) as input, while model 2 was trained using real X-rays. In total, model 1 was trained and validated using 235 images from three separate centers. Model 2 was trained and tested using 1323 images from a single center.

**Results:**

The performance of the model using DDRs as input reached a peak-signal-to-noise ratio (PSNR) of 15.61 and a structural similarity index measure (SSIM) of 0.782 during external validation. The second model, using real X-rays as input, attained a PSNR of 14.69 and an SSIM of 0.717 upon internal validation.

**Conclusions:**

At the present stage, the synthetic cranial tomography scans generated as part of this study show promise but do not seamlessly correspond to ground-truth CTs. However, this proof-of-concept study is the first to derive such artificial cranial images using deep learning and can serve as a starting point for further investigation.

**Key Points:**

***Question*** *Cranial computed tomography involves radiation, logistical challenges, and access is limited in rural areas. Generating synthetic CT images with deep learning could address these challenges*.

***Findings*** *Two deep-learning models were trained to produce CT images from radiographs. Reconstruction from DRRs is promising, but using real X-rays remains more challenging*.

***Clinical relevance*** *As a proof-of-concept, the models’ exact clinical relevance remains to be defined. The proposed approach may broaden access to tomographic neuroimaging, reduce radiation, and enhance intraoperative and maybe even diagnostic support, potentially improving outcomes in neurosurgery and neuro-critical care*.

**Graphical Abstract:**

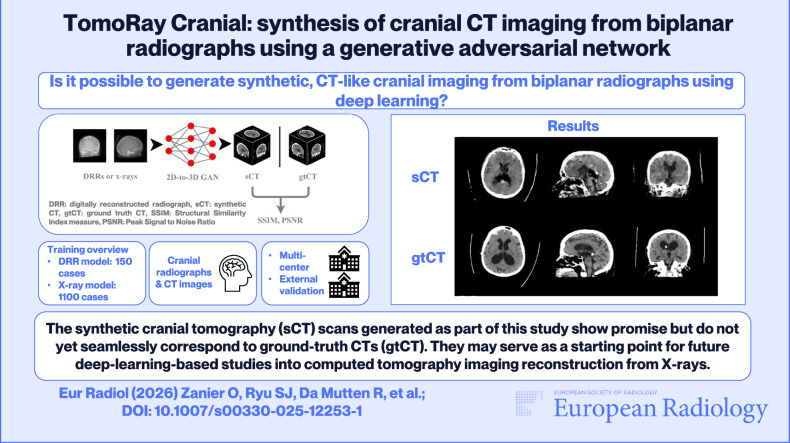

## Introduction

In neurotrauma or vascular emergencies, repeated CT imaging is often necessary, particularly in unconscious patients, that  only allow for limited neurological examination [1, 2]. Repeated imaging allows for tracking of, e.g., hematomas, cerebrospinal fluid status, midline shift, and herniation [3, 4]. Still, CT imaging incurs relevant logistical problems, in particular for hemodynamically unstable patients or those attached to multiple life-supporting systems such as ventilators, hemodialysis, ECMO, and multiple monitors. Local options for CT acquisition in the intensive care unit or trauma bay, such as mobile CT or even mobile MRI, have been explored [5]. However, when applied to such a critical care patient population, these methods have drawbacks, such as radiation exposure, costs, and the need to position patients in a supine position without head elevation. Moreover, many centers worldwide do not have access to such niche technologies.

Similarly, during neurosurgical procedures ranging from insertion of an external ventricular drain (EVD) to resection of brain tumors, repeated 3D imaging would be beneficial, given that it is quickly accessible and sufficiently accurate [6]. Intraoperative CT (especially cone-beam CT) and intraoperative MRI have been adopted in many centers [7–9]. Still, these modalities are associated with high acquisition and maintenance costs in addition to logistical challenges and are, therefore, typically only used once during surgery [5, 7, 8].

It has been shown that it is possible to produce thorax CT-like imaging using biplanar chest X-rays as input for a deep learning model [10]. No cranial applications of such an algorithm have been reported so far. As the brain parenchyma is completely encased by the skull, three-dimensional soft tissue window reconstruction from cranial X-rays is more challenging compared to chest X-rays with better soft tissue translucence. However, such a 2D-to-3D reconstruction method could prove to be effective whenever obtaining a 3D image of the skull is not feasible or practical. This study presents a preliminary effort to create artificial, cranial CT scans from biplanar radiographs of adult neurosurgical patients using deep learning. It sets a starting point for further studies to explore the day-to-day clinical utility of such an algorithm.

## Materials and methods

### Ethical considerations and patient consent

Patient data were handled in accordance with the ethical standards outlined in the Declaration of Helsinki and its amendments. The use of this data received approval from the institutional review boards in Zurich (IRB, Cantonal Ethics Committee Zürich, BASEC 2023-00689) and Daejeon Eulji University Hospital (No. 2023-12-012). For data collected in Bologna, ethical approval was granted by the ethics committee of the greater area of Emilia-Romagna (No. 94-2025-OSS-AUSLBO).

### Data collection

#### Model 1: digital reconstructed radiograph (DRR)-based

Three datasets from different centers were considered for the development and evaluation of model 1. The Zurich dataset contained patients who underwent shunt implantation or shunt revision surgery for hydrocephalus between September 2020 and December 2022 and was retrospectively evaluated. The inclusion criteria were the availability of post-interventional computed tomography and X-ray imaging of the skull obtained up to 48 h after shunt insertion in adult (age 18 or over) patients. Imaging from patients with shunt placement was used, as this patient group routinely receives postoperative X-rays. The availability of corresponding X-ray imaging is intended to allow for visual comparison with the DRRs generated in this study and for future model training using real X-rays as input, but these X-rays have not yet been applied in the training of model 1. Patients with insufficient imaging (no corresponding X-ray within 48 h) were not considered. Different etiologies of hydrocephalus were included in the dataset. The Zurich dataset comprises CTs from a total of 114 examinations.

The openly available CQ-500 dataset consists of retrospectively collected head CT scans that were acquired at different institutions in New Delhi [11]. Pathologies included intraparenchymal hemorrhages, subdural hemorrhages, epidural hemorrhages, and subarachnoid hemorrhages, as well as calvarial fractures. Follow-up scans for some of the patients had to be manually excluded to ensure that only one CT scan for each patient was kept in the final dataset. Additionally, several poor-quality scans (significant distortion and/or very low resolution) had to be omitted. 71 CT scans were kept in the final dataset and used for model training.

A third dataset contained retrospectively collected CT imaging of 50 normal-pressure hydrocephalus patients after shunt placement from Bologna. Images were retrospectively collected from the years 2016–2019.

Since the training of our machine learning model requires standardized data, and corresponding X-rays and CT scans are difficult to obtain (X-rays were available only for the Zurich dataset), DRRs were used as input for the training of model 1. Furthermore, there is heterogeneity in how anatomical structures align between X-rays and CTs. This poses a challenge for supervised model training. DRRs allow for the generation of X-ray-like images from 3D-CTs with relatively high accuracy and thus can serve as a substitute for genuine X-rays. CT scout images could have been used as input, but DRRs, in theory, resemble real X-rays more closely due to simulating ray projection from an imaginary probe [12]. Still, DRRs do not correspond exactly to actual X-rays, which is why we explored using real X-rays for model 2. All DRRs were derived using the Plastimatch software [13, 14].

**Fig. 1:**
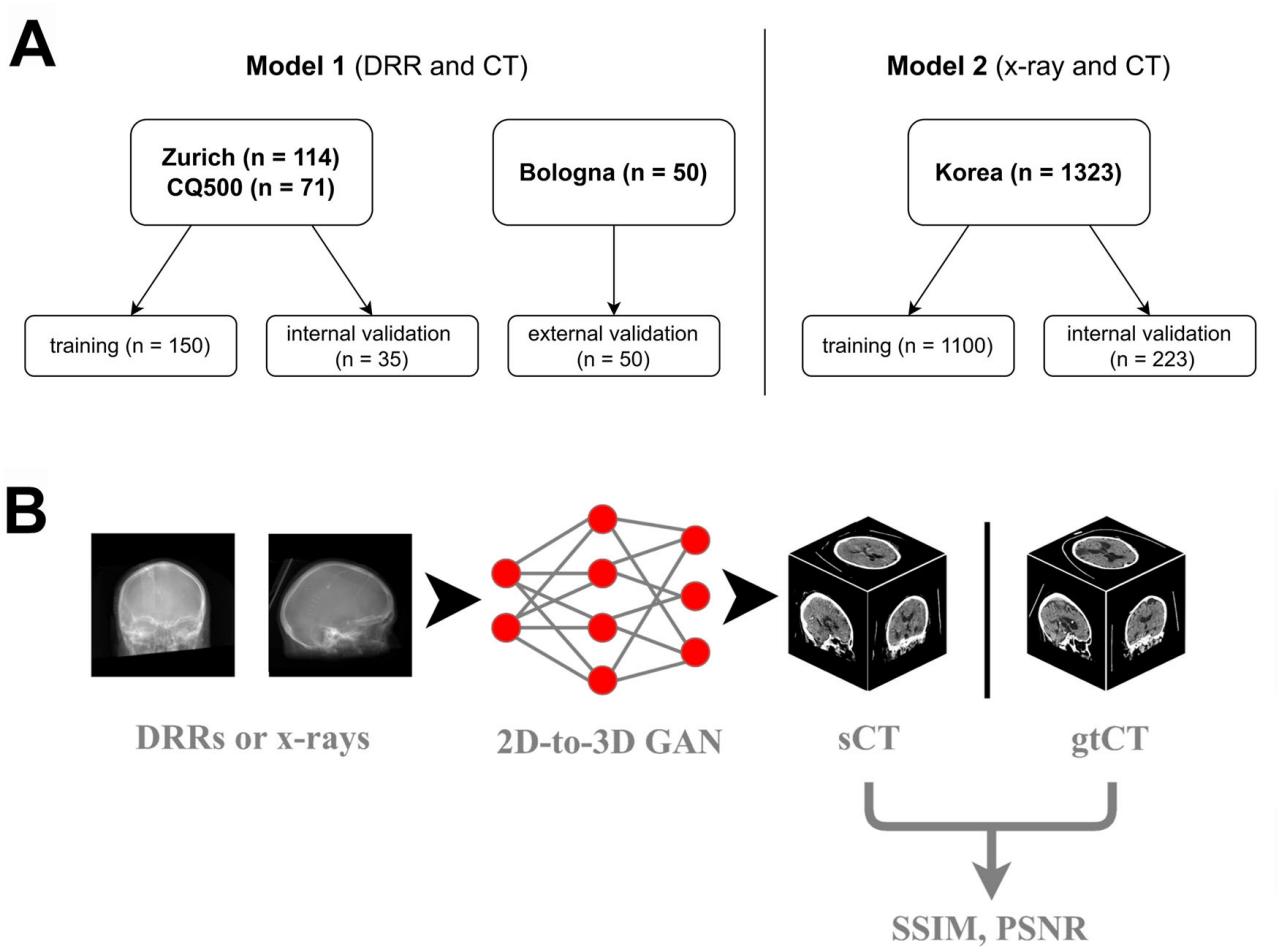
Study overview. **A** Overview of data allocation in this study. **B** Schematic representation of model application to new data and performance evaluation using image similarity metrics. Example images were taken from the internal validation set of model 1. gtCT, ground truth CT; sCT, synthetic CT generated by GAN; PSNR, peak signal to noise ratio; SSIM, structural similarity index measure; DDR, digitally reconstructed radiograph; GAN, Generative adversarial networks

Table 2 provides an overview of the patient characteristics, imaging information, and disease characteristics for the Zurich and Bologna datasets. Figure 1A and Table 1 display the attribution of the aforementioned data into training and validation datasets.

#### Model 2: X-ray based

1323 images from the Daejeon Eulji University Hospital were used for training and internal evaluation of model 2. All images in this dataset were retrospectively collected from the years 2015 to 2022. The inclusion criterion was the availability of paired computed tomography and X-ray imaging of the skull in adult (older than 18 years) patients. Fluoroscopy imaging instead of X-rays was also included after inverting the grayscale to make the images appear akin to X-rays. These inverted images resemble X-rays enough to justify inclusion.

Care was taken to prevent data leakage between the datasets. Table 3 presents an overview of the pathologies found in the dataset used for training of model 2.

### Preprocessing, model development, and validation

#### For both models 1 and 2

In a first step, the de-identified CT images were converted from DICOM imaging into NIfTI format. To get a reliable ground truth CT (gtCT) dataset, we used a rigid transformation algorithm that aligned all imaging with a standardized CT scan, calculated by Rorden et al from 35 high-resolution CT scans without parenchymal pathology [15]. Next, all CT scans were resampled to isotropic voxel spacing (1 × 1 × 1 mm^3^), and the resolution of all images in the dataset was homogenized to 256 × 256 × 256 voxels. Notably, this only served as a starting point, and images were later automatically resized prior to model training to the image dimensions specified in the training parameters. These specified dimensions correspond to the model’s output layer size and, therefore, resolution, and were carefully chosen to utilize the maximum computational capacity of the respective training machines. Hounsfield windowing for brain parenchyma was applied (window center 40 HU, window width 40 HU). For both models, the architecture of the generative adversarial model (GAN) first described in X2CT-GAN was used [10]. This model architecture proved successful in reconstructing three-dimensional CTs from chest radiographs, providing a suitable framework for the task at hand. Other model architectures for the reconstruction of three-dimensional images from a two-dimensional input exist, however, they do not use deep learning, source code is not readily available, or the model architecture would need to be significantly amended for the purposes of our study [16, 17].

This study’s focus was not to explore a specific intracranial pathology but merely to investigate the feasibility of 2D to 3D X-ray to CT-like imaging conversion. Thus, no systematic assessment of pathology recognition in the synthetic CTs (sCTs) was conducted.

A schematic representation of the model application and evaluation is depicted in Fig. 1B.

#### Model 1 (DRR-based)

Using the Plastimatch software, the DRRs from the CT scans were derived to be used as model input during the training process [13]. This was carried out prior to Hounsfield windowing of the CT scans using the “plastimatch drr” command [18]. The dimensions of the DRRs generated were 320 × 320 pixels.

Model 1 was trained on 150 CT examinations to generate sCTs using the biplanar DRRs as input. Model parameters were randomly initialized, and manual hyperparameter tuning was performed wherever necessary. The training set consisted of the CTs and their corresponding DRRs. The final GAN was trained with a learning rate of 0.0001 for 150 epochs before decreasing the learning rate for 30 more epochs using a lambda learning rate scheduler. The resolution of the output images was 192 × 192 × 192 voxels.

After completing the training process, the final model was assessed on both internal and external validation sets, which contained images of 35 and 50 patients, respectively.

For model 1, the code was executed on a Windows operating system running Python 3.10.6 and PyTorch 1.12.1 for CUDA 11.6 [19, 20].

**Fig. 2:**
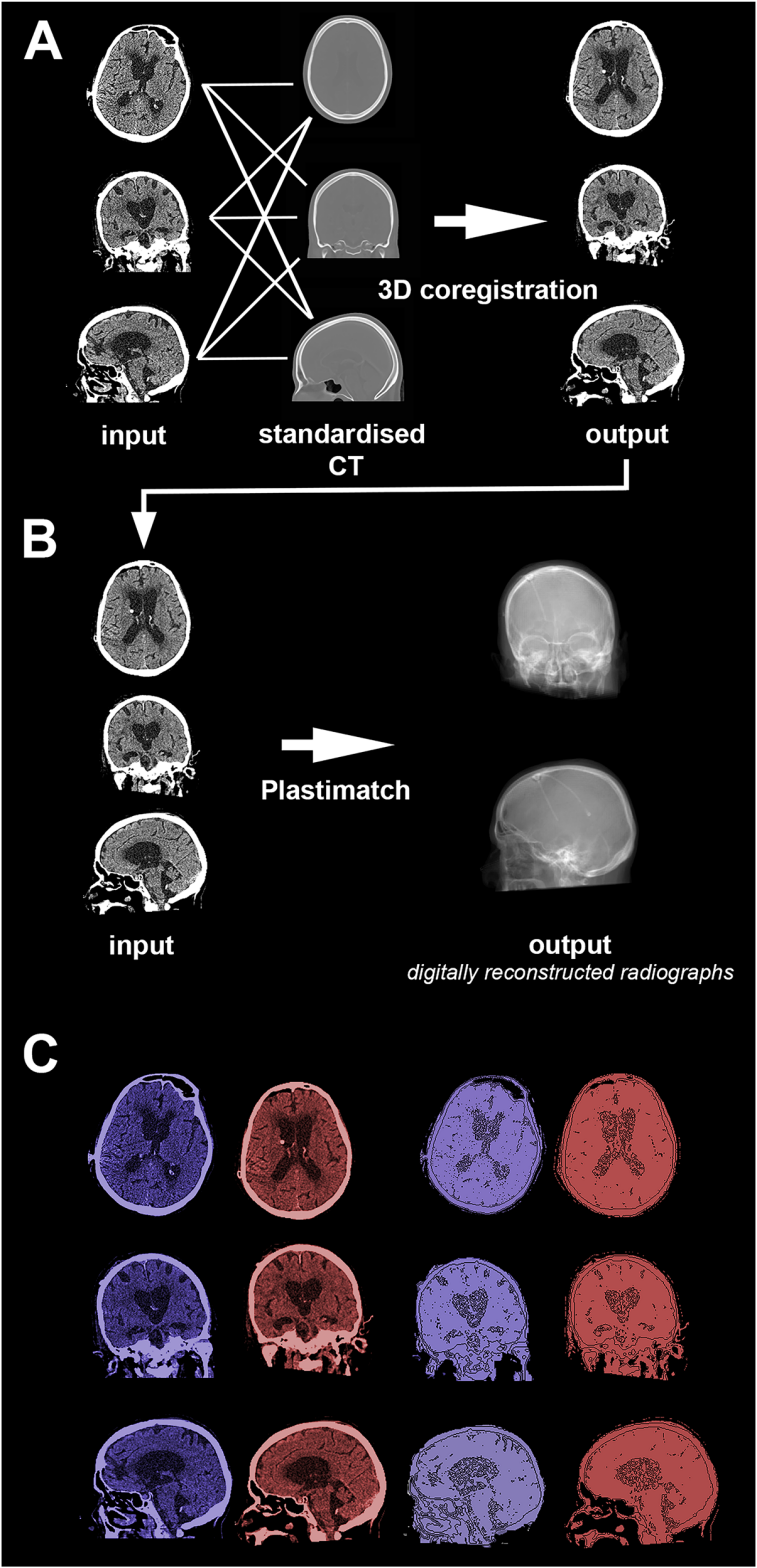
Preprocessing for model 1. **A** Step1: registration of a windowed CT scan to a standardized CT scan. **B** Step 2: creating a digitally reconstructed radiograph from the now standardized CT scan. **C** Comparison of input CT (blue) vs same CT after coregistration (red)

Preprocessing for model 1 is schematically depicted in Fig. 2.

#### Model 2 (X-ray based)

In contrast to model 1, real X-rays were used in the training of model 2. If needed, the X-rays were adjusted manually to ensure that they all had the same orientation. Based on the distribution of pixel intensities, any fluoroscopy images in the dataset were automatically recognized, and grayscale values inverted.

A YOLOv8l was trained on 196 hand-labeled X-rays (98 a.p., 98 lat.) from the Zurich dataset to detect and extract the neurocranium (specifically, frontal, ethmoid, sphenoid, parietal, temporal, and occipital bones) [21]. It was then evaluated on an internal validation set containing 30 images (15 a.p., 15 lat.) using average precision (AP50 and AP50-95) [22]. Its performance was not quantitatively assessed on the Korea dataset. Before inputting any images into YOLOv8 for training and inference, the X-rays were padded with black pixels along their shorter sides to form square images. Extraction of the neurocranium was conducted on the full-resolution images. Due to computational constraints, the extracted regions of interest were then resized to 512 × 512 pixels before co-registration to normalized skull X-rays. These normalized X-rays are two DRRs (a.p. and lat) that were acquired from the standardized CT scan using Plastimatch [13, 15]. In summary, the preprocessing of all X-rays from the Korea dataset encompassed detecting the neurocranium on the X-rays using a YOLOv8l [21], and subsequent rigid registration of the cropped-out skull to the previously mentioned two standardized DRRs [23].

These two steps allowed us to partially account for the differences in absolute sizes, rotation, or translation of any structure in X-rays compared to the gtCT.

The Korea dataset, used for model 2 training and validation, was split at the patient level. The final training set consisted of 1200 CT scans with their paired X-rays (or inverted fluoroscopy images). Model 2 was trained for 150 epochs using a learning rate of 0.00025, and the learning rate was decreased using a lambda learning rate scheduler for 50 epochs thereafter. Only minimal hyperparameter tuning was performed. Output sCT resolution was 128 × 128 × 128 voxels.

An internal validation dataset, including 123 examinations, was utilized for the evaluation of model performance.

For model 2, all code was executed on a Windows operating system running Python 3.10.13 and PyTorch 2.1.1 for CUDA 12.1 [19, 20].

**Fig. 3:**
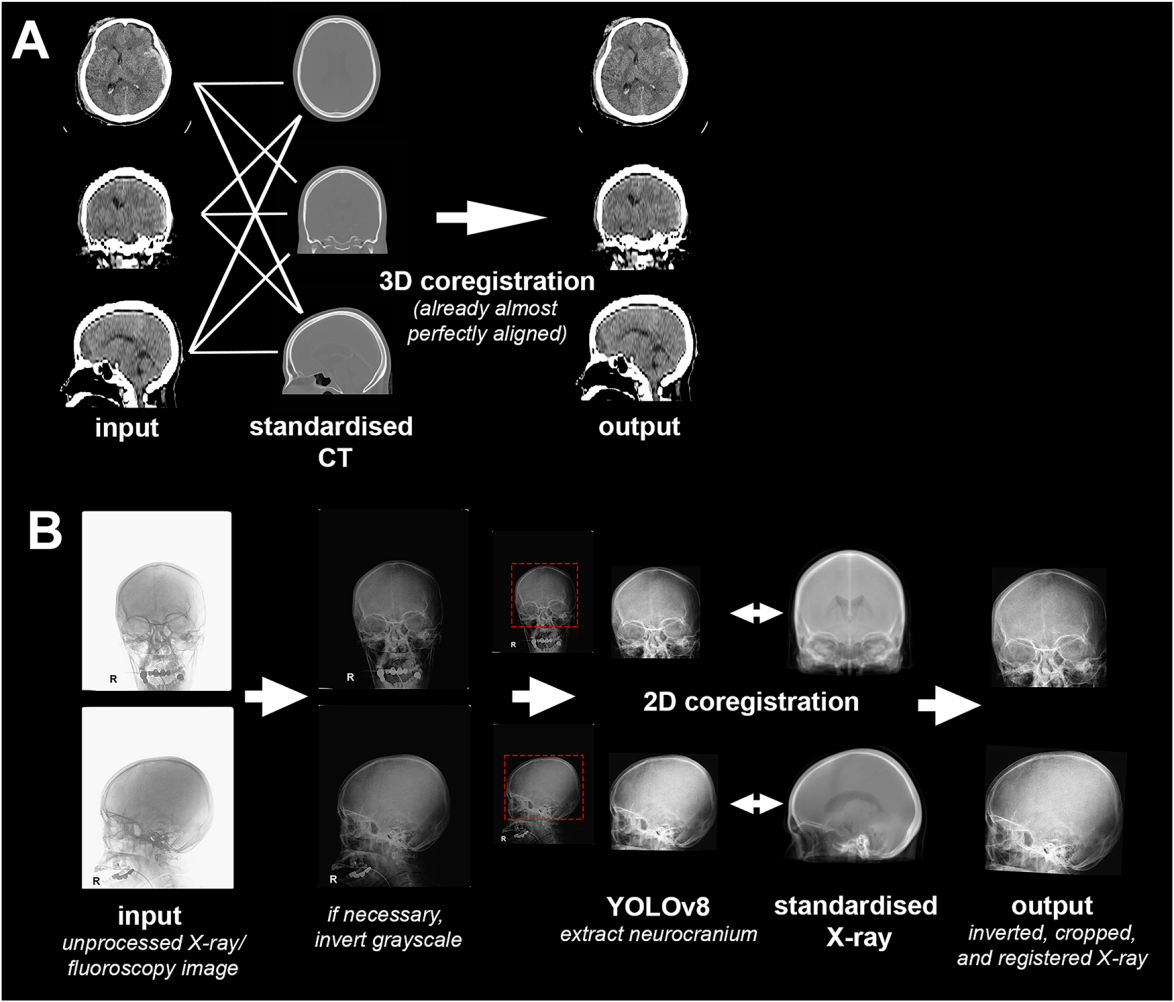
Preprocessing for model 2. **A** Step1: registration of a windowed CT scan to a standardized CT scan. **B** Steps 2, 3, and 4: Inversion of grayscale (if necessary), extraction of neurocranium, and rigid registration of patient X-rays to standardized X-rays

Preprocessing for model 2 is schematically displayed in Fig. 3.

### Metrics and evaluation

Improvements in the alignment of patient CTs with the standardized cranial CT through coregistration were assessed using DICE Score, average symmetric surface distance (ASSD), center of mass (COM), and principal axis (PA). The DICE score quantified the volumetric overlap between binary cranial masks from the patient’s CT and the standardized CT, while ASSD measures the average shortest distance between the borders of the two corresponding binary masks. COM represents the mean voxel position (geometric center) of all voxels, and PA was derived from principal component analysis of voxel coordinates within the binary masks, describing the dominant spatial orientation of the cranial volume. The dominant orientation typically corresponds to the occipitofrontal direction. In contrast to the other alignment metrics, PA determination for cranial CTs is relatively error-prone, particularly for patients with near-spherical skulls (similar in size across all three dimensions). Accordingly, PA should be considered a qualitative metric (improvement vs. no improvement) rather than a precise quantitative metric.

The quality of the reconstructed sCTs from models was evaluated using the peak signal-to-noise ratio (PSNR) and structural similarity index measure (SSIM). SSIM and PSNR are distinguished image quality metrics and have been extensively described in previous studies [10, 24–26]. Average precision, as used to evaluate the neurocranium extraction model used in standardizing input X-rays for model 2, is an established metric for object detection tasks [27]. In an effort to assess model 1’s capacity to capture basic anatomical relationships, skull width, skull length, cephalic index (maximum skull width divided by maximum skull length), and Evans index were compared between gtCTs and sCTs. Additionally, the overlap of binary skull masks was investigated using the DICE Score (Supplementary Fig. 3).

## Results

### Patient cohort

#### Model 1 (DRR-based)

Model 1 was trained using imaging data from 150 CT examinations (Zurich and CQ 500), and its performance was evaluated on a total of 85 scans (Zurich and CQ500 for internal, Bologna for external validation). The cases in the internal validation dataset were randomly selected to obtain an equal distribution. The most common hydrocephalus etiologies from the Zurich dataset were bleedings, normal pressure hydrocephalus, and tumors in descending order.

#### Model 2 (X-ray based)

1200 examinations were used in training, while 123 scans were withheld and served as a validation dataset (all from Korea). The selection of scans for the internal validation dataset was random to ensure an equal distribution. No information on patient demographics and absolute counts of pathologies was available.

Detailed patient characteristics and information on the dataset compositions are provided in Tables 1–3.

**Table 1:** Dataset composition for models 1 and 2

Model 1	Training (*n* = 150)	Internal validation (*n* = 35)	External validation (*n* = 50)	Total (*n* = 235)
Zurich	94 (62.7%)	20 (57.1%)	-	114 (48.5%)
CQ500	56 (37.3%)	15 (42.9%)	-	71 (30.2%)
Bologna	-	-	50 (100%)	50 (21.3%)
**Model 2**	**Training**	**Internal validation**	**-**	**Total**
Korea	1200 (90.7%)	123 (9.3%)	-	1323 (100%)

The data division into training and internal validation sets was random to ensure unbiased evaluation and effective training

**Table 2:** Patient, disease, and imaging characteristics of the Zurich and Bologna datasets

	Zurich	Bologna
Demographics		
Age (mean ± SD) [years]	63.30 ± 17.38	75.94 ± 4.17
Male gender n (%)	53 (46.49%)	26 (52.0%)
Patient size [cm]		
Male	173 ± 22	-
Female	162 ± 8	-
*nr unavailable*	*2 (0.2%)*	*50 (100%)*
Patient weight [kg]		
Male	76.6 ± 21.0	-
Female	65.1 ± 16.3	-
*nr unavailable*	*2 (0.2%)*	*50 (100%)*
Pathology		
Bleeding (SAB, ICB, etc.) excl. trauma	49 (42.6%)	-
Normal pressure hydrocephalus	29 (25.2%)	50 (100%)
Tumor/metastasis (incl. vHL & NF)	16 (13.9%)	-
Trauma	9 (7.8%)	-
Malformations (Chiari, cysts, etc.)	4 (3.5%)	-
Others (ischemia, meningitis, etc.)	3 (3.5%)	-
Unavailable	4 (3.5%)	-
Shunt type		
Ventriculoperitoneal (VP)	112 (98.25%)	50 (100%)
Ventriculoatrial (VA)	2 (1.75%)	
Revision surgery		
Yes	24 (21.05%)	-
No	90 (78.95%)	50 (100%)
CT manufacturers		
Siemens/Siemens Healthineers	112 (98.2%)	41 (82.0%)
Philips		6 (12.0%)
GE Medical		3 (6.0%)
Unknown	2 (1.8%)	
Slice thickness [mm]		
	0.799 ± 0.008	2.832 ± 0.575
Pixel spacing [mm]		
*x*-axis	0.473 ± 0.039	0.473 ± 0.039
*y*-axis	0.473 ± 0.039	0.473 ± 0.039

*SAB* subarachnoid hemorrhage, *ICB* intracerebral hemorrhage, *vHL* von Hippel Landau syndrome, *NF* neurofibromatosis type 1 and 2 Results are reported as mean ± SD for continuous and *n* (%) for categorical variables

**Table 3:** Disease characteristics from the Korea dataset

Pathology	Count
Intracerebral hematoma	633 (47.9%)
Subdural hematoma	582 (44.0%)
Subarachnoideal hemorrhage	530 (40.1%)
Skull fracture of any kind	368 (27.8%)
Epidural hematoma	334 (25.2%)
No finding	87 (6.6%)
Unknown	9 (0.7%)

Up to three of the most immediate diagnoses per patient, based on the radiology report, are included in the analysis

### Model performance

Tables 4 and 5 present an overview of the model performances for models 1 and 2, respectively.

**Table 4:** Model 1 performance on the training and holdout set

	PSNR 3D (dB)	SSIM
Training (*n* = 150)		
mean ± SD	21.90 ± 1.18	0.873 ± 0.024
median (IQR)	21.87 (21.22–22.53)	0.876 (0.855–0.890)
Internal validation (*n* = 35)		
mean ± SD	16.68 ± 1.01	0.799 ± 0.029
median (IQR)	16.39 (15.90–17.33)	0.806 (0.778–0.820)
External validation (*n* = 50)		
Mean ± SD	15.61 ± 1.02	0.782 ± 0.030
Median (IQR)	15.63 (14.92–16.32)	0.778 (0.766–0.804)

Final values reported are the mean and median over the whole dataset

*SD* standard deviation, *IQR* interquartile range, *PSNR* peak signal to noise ratio, *SSIM* structural similarity index measure

**Table 5:** Model 2 performance on the training and holdout set

	PSNR 3D (dB)	SSIM
Training (*n* = 1200)		
mean ± SD	17.13 ± 1.05	0.803 ± 0.033
median (IQR)	17.06 (16.42–17.69)	0.806 (0.782–0.821)
Internal validation (*n* = 123)		
mean ± SD	14.69 ± 0.85	0.717 ± 0.037
median (IQR)	14.77 (14.25–15.23)	0.720 (0.696–0.741)

Final values reported are the mean and median over the whole dataset

*SD* standard deviation, *IQR* interquartile range, *PSNR* peak signal to noise ratio, *SSIM* structural similarity index measure

#### Model 1 (DRR-based)

Registration: the cranial structures in the CQ500 dataset were already well aligned and, therefore, not included in this analysis. Overall, alignment with the standardized head CT for the 164 images from the Zurich and Bologna datasets on average improved by 1.7% in their DICE Score. ASSD improved by a mean of 8.3% and the distance between COMs decreased by 25.2% on average. The error between principal axes dropped by 5.9%. A more detailed overview is provided in Supplementary Table 1, and Supplementary Fig. 2 provides an example illustrating the improvement achieved by coregistration.

Image quality: performance evaluation at internal validation yielded a PSNR of 16.68 ± 1.01, and the SSIM reached a score of 0.799 ± 0.029.

During external validation, a PSNR of 15.61 ± 1.02 was achieved. The SSIM amounted to 0.782 ± 0.030.

**Fig. 4:**
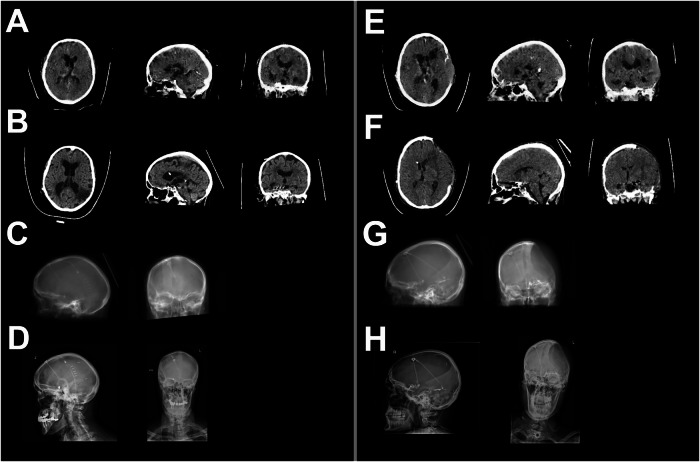
Example of model 1 output on the internal validation dataset. **A**–**D** Good reconstruction example. **E**–**H** Less successful reconstruction. **A**, **E** sCTs generated by the GAN. **B**, **F** Corresponding gtCT. **C**, **G** Digitally reconstructed radiograph used as model input. **D**, **H** Actual X-rays from the shunt series for comparison with DRRs (not used for reconstruction). sCT, Synthetic CT; gtCT, Ground truth CT; GAN, Generative adversarial networks; DRR, Digitally reconstructed radiographs

**Fig. 5:**
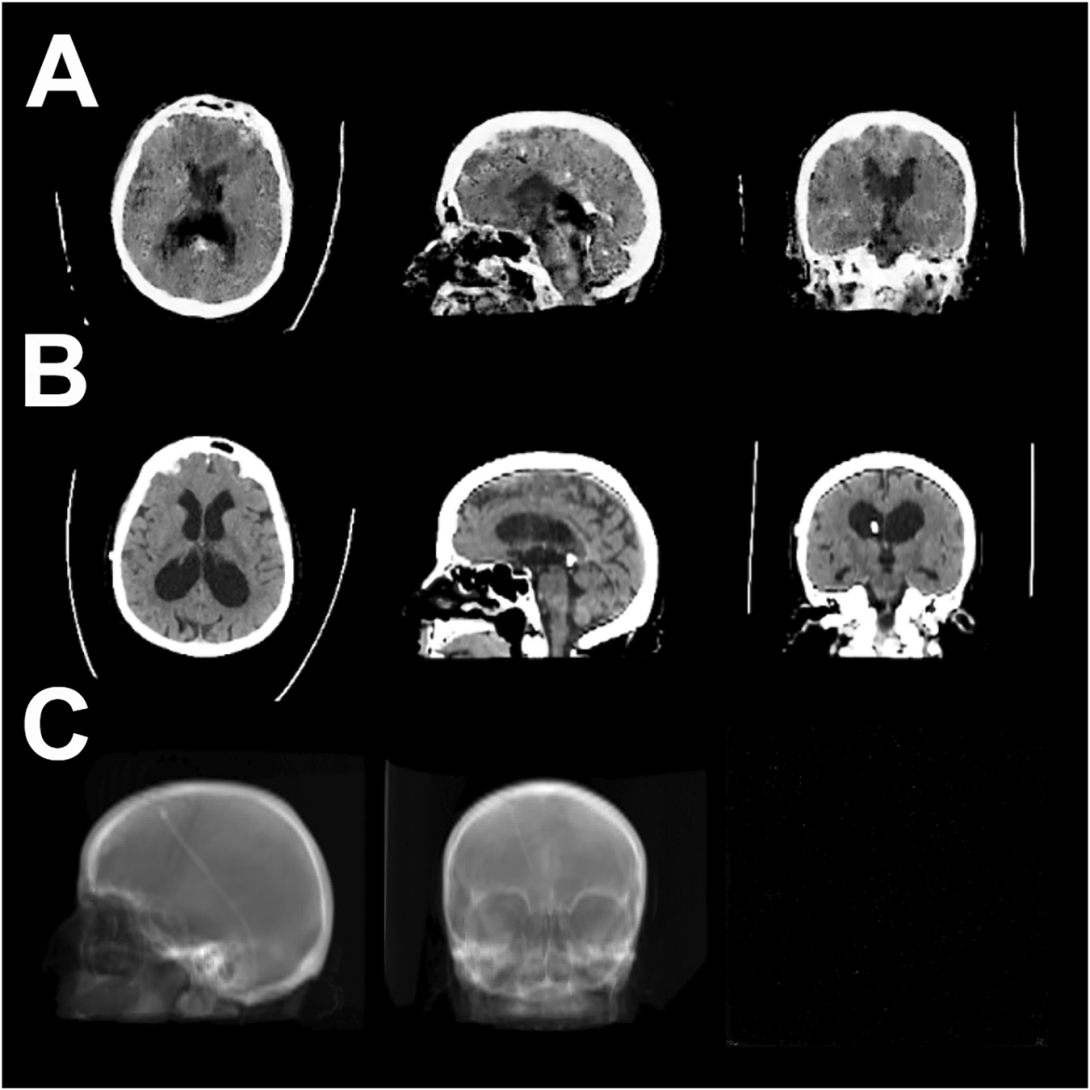
Example of a good reconstruction by model 1 on the external validation dataset. **A** sCTs generated by the GAN. **B** Corresponding gtCT. **C** Digitally reconstructed radiograph was used as model input. No corresponding X-rays were available for the CT scans in the external validation dataset. sCT, Synthetic CT; gtCT, Ground truth CT

Examples of the imaging generated by model 1 are depicted in Figs. 4 and 5, for cases taken from the internal and external validation sets, respectively. Supplementary Fig. 1 shows underperforming cases taken from the external validation dataset.

Anatomical correlations: the overlap of skull masks generated from gtCTs and reconstructed CTs yielded a DICE Score of 0.68 and 0.59 on the internal and external validation sets, respectively (Supplementary Fig. 4).

Both maximum skull diameter and maximum skull width showed moderate to strong correlations between ground truth images and reconstructed images, achieving Pearson correlation coefficients of 0.60 and 0.89, respectively. With a 0.16 correlation, Evan’s index only showed a low correlation at external validation (Supplementary Fig. 5).

#### Model 2 (X-ray based)

Internal validation of the the YOLOv8 model used to extract the neurocranium from the cranial X-rays resulted in a mAP50 of 0.989 and a mAP50-90 of 0.798.

At internal validation of model 2, a PSNR of 14.69 ± 0.0.85 and an SSIM of 0.717 ± 0.034 were recorded.

**Fig. 6:**
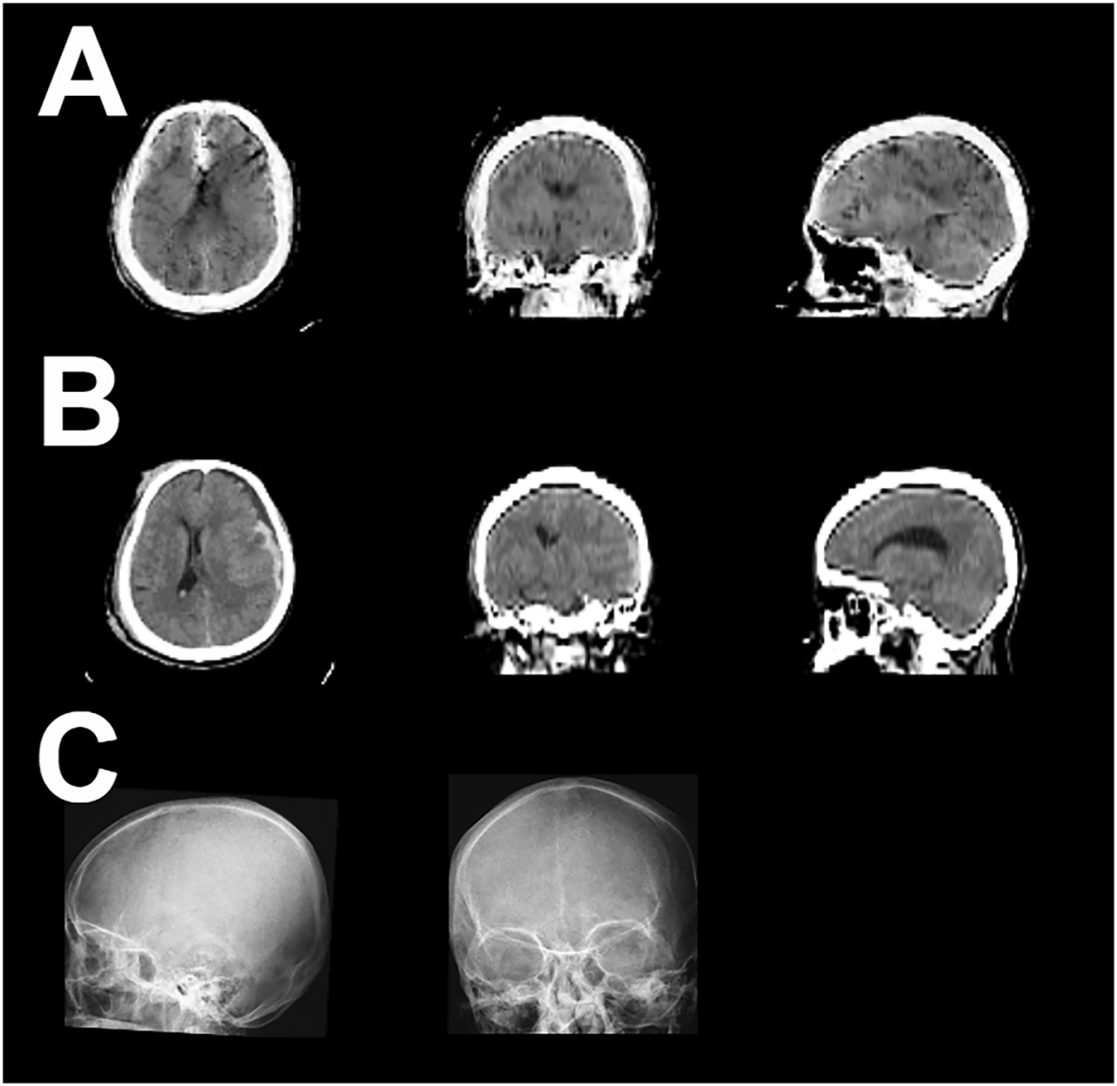
Example of model 2 output on the internal validation dataset. **A** sCTs generated by the GAN. **B** Corresponding gtCT. **C** Input X-ray. sCT, Synthetic CT; gtCT, Ground truth CT; GAN, Generative adversarial networks

Figure 6 shows an sCT generated by model 2 for a case from the holdout set.

## Discussion

With multi-center data from over 1500 patients, two generative adversarial networks (GANs) for the conversion of radiographs into sCTs were developed and validated. One model used DRRs as input, while the second GAN can process actual biplanar X-rays. Performance in terms of similarity to gtCT was moderate, especially for model 2. Although proper working models were derived in this study, in their current preliminary state, the synthetic images are still lacking behind real CT scans in terms of objectiveness and resolution. Their added value in clinical practice needs to be critically examined. However, to the best of the authors’ knowledge, this is the first study in which synthetic head CTs from both biplanar DRR, as well as real X-rays, were generated using deep learning.

We assessed the improvements in the standardization of CT image orientation for model 1 achieved through registration. DICE Score, ASSD, COM distance, and PA all improved after coregistration, indicating an improvement in overlap, boundary match, centering, and rotational alignment after coregistration. The improvements for the metrics ranged between 1% and 25% and might appear marginal, however, most CTs in the dataset were already well aligned to begin with. The coregistration step primarily addresses minor inconsistencies in the images’ orientation and additionally acts as a failsafe for the few images with severely misaligned cranial structures.

Generation of sCT from X-rays could be highly beneficial, reducing time, cost, and radiation exposure to the patient. After ventriculoperitoneal shunt surgery, CT scans, as well as shunt series, are routinely carried out to assess correct placement, as well as sufficient reduction in size of the ventricles [28, 29]. Radiography alone is not sufficient to enable three-dimensional evaluation of proper shunt placement. An enhanced version of the model proposed in this study could, therefore, enable neurosurgeons to use the imaging from the shunt series to create three-dimensional information without the need to expose the patient to any further radiation or even allow for intraoperative validation of correct catheter positioning.

Both the Zurich and Bologna datasets contain images from hydrocephalus patients exclusively. This was done intentionally to reduce heterogeneity in the imaging data, as our study presents a preliminary approach to 2D-to-3D cranial radiograph imaging conversion. Hydrocephalus patients were selected because they are among the few neurosurgical cases that routinely undergo both CT and X-ray imaging within a short timeframe. This design allows for the comparison of DRRs with the real X-rays and will enable us to refine our model using real X-rays as input instead of DRRs in future work. We acknowledge that only including hydrocephalus patients for the Zurich and Bologna datasets inherently limits the generalizability of our findings to other pathologies. Nevertheless, we believe that, at least until broader applicability is demonstrated, the focus should lie on a single pathology. This approach will allow for the assessment of how well that pathology is represented in the reconstructed imaging without introducing bias from mixed-pathology training. Once this has been established, the feasibility of more generalizable models should be explored in future work. Using real radiographs instead of DRRs is essential to create a clinically applicable model, but the lack of available X-ray data acquired in a standardized fashion hinders development. Significant heterogeneity in patient positioning and image magnification during X-ray acquisition impedes efficient training of machine learning models [30, 31]. Even though the proposed X-ray pre-processing method aims to counteract this by extracting the neurocranium and co-registering it to a standardized DRR, its impact is partially reflected by the significantly lower quality of the sCT generated by model 2. A recent study showed that using DRRs acquired at an angle other than 90° can negatively impact CT reconstructions for spinal radiographs [32]. While some degree of heterogeneity is inevitable in the clinical workflow, a more standardized technique for radiograph acquisition could help reduce variability and may  be beneficial for training a model using real X-rays. DRRs, used as a proxy for real X-rays, are not able to fully match real radiographs in terms of resolution and visualization of subtle anatomical structures. Nonetheless, they provide a more readily available and commonly accepted alternative for proof-of-concept studies [10, 12].

GANs are capable of creating realistically looking artificial images, perform image, as well as text-to-image translation, and enhance image resolution [33–35]. Cross-modality transfer to CT using machine learning has been applied, for example, from MRI to sCT or cone beam CT to spiral CT [36, 37]. However, all these studies aimed at transforming 3-dimensional imaging from one modality into another 3-dimensional modality. Xingde Ying et al created a GAN that allowed the transformation of biplanar chest radiographs into a synthetic chest CT [10]. By applying their GAN architecture, after adjusting the parameters to the needs of our task, preliminary models allowing transformation of biplanar head radiographs into sCT of the cranium were derived in this study. Similar adaptations have already been explored for spinal imaging [26, 32]. 2D-to-3D sCT reconstruction is not an entirely new field of research, but while studies before 2018 mainly focused on statistical methods like statistical shape models or iterative reconstruction algorithms, deep learning has become the primary driver since [38]. One of the earliest methods was digital tomosynthesis, which dates back to the 1970s, and FDA approval for breast cancer screening was granted in 2013, over 40 years later [39]. This further illustrates the lengthy development process that novel imaging modalities undergo, with deep learning-based reconstructions only being at the beginning of this process. So far, the most investigated anatomical region for CT reconstruction is the chest, with only a smaller fraction of studies focusing on cranial structures. More importantly, while most studies reported validating their models’ performances, only 22% of all studies performed an external validation [38]. This lack of adequate validation limits generalizability and, therefore, clinical application. It further underlines the necessity for thorough external validation, as performed in this study. Regarding quantitative performance, model 1 achieved an SSIM of 0.78 upon external validation. This roughly aligns with the range observed in comparative studies for chest X-rays, where an SSIM of 0.72 was achieved, and for the spine, where an SSIM of 0.47 was reported [10, 32]. However, these comparisons should be interpreted with caution, as, besides potential methodological differences, both studies included images with significantly higher amounts of soft tissue, whereas cranial CTs typically feature larger areas of background.

In the far future, 2D-to-3D sCT reconstruction models could be implemented in the clinical workflow of radiologists, neurologists, neurosurgeons, and intensive care specialists, among other stakeholders. For parenchymal diagnostics and guidance during treatment, tomographic reconstructions are considered an absolute necessity. While H. Cushing was able to localize large meningiomas and W. Dandy performed ventriculographic studies for the identification of brain tumors using X-rays only [40, 41], today’s requirements necessitate much more granular judgment of bone and parenchyma in 3D. Generally, X-ray and fluoroscopic imaging cannot fulfill these prerequisites. There are specialized intraoperative 3D-fluoroscopy machines that can deliver 3D fluoroscopically generated CTs [42]. However, due to their high acquisition and maintenance costs, their global rollout is limited. In the future, a model similar to the ones trained in this study could derive a three-dimensional sCT from such fluoroscopic imaging without the need for any additional equipment. Due to the limited spatial information and anatomical resolution in X-rays, deep-learning-based reconstructions of tomographic imaging will likely not match the actual CTs in their quality. Thus, it is not realistic to replace CTs in all clinical scenarios. But, in circumstances where radiographic imaging on its own is proven to be insufficient and tomographic imaging might be considered excessive, a deep-learning model, like the one proposed in this study, could find its place. Example indications may include diagnostics of traumatic brain injuries in low-resource settings or evaluation of correct extra-ventricular drain placement directly from postoperative (or even intraoperative) radiographs. Moreover, postoperative pneumocephalus after burr hole surgery might be assessed. Further studies should explore the application of an enhanced version of the proposed models to such specific clinical scenarios to assess their clinical usefulness.

In an initial attempt to evaluate how well the model captures fundamental anatomical relationships, we compared skull width, skull length, and Evans index for model 1 between synthetic and ground-truth CTs. Calvarial measurements showed strong correlations, whereas the correlation for the parenchymal Evans index was low. This finding is not unexpected, as biplanar 2D radiographs provide reliable information about skull morphology but convey only limited insight into intracranial structures. Future studies will be needed to determine whether and to what extent reconstructed radiographs can accurately represent skull fractures or intracranial pathologies. While this study provides an idea of the potential advantages of X-ray to CT conversion, there are some inherent limitations to it. Despite being state-of-the-art, the metrics applied for quantitative evaluation of image reconstruction tasks do not necessarily correspond to visual acuity of the synthetic imaging [10, 32, 43]. Blurrier imaging can, under certain circumstances, achieve better image quality performance scores than visually accurate images [44]. Nevertheless, due to a lack of alternatives, we still opted to use these metrics. An additional caveat, especially for models intended to be clinically applied by physicians in the future, is that GANs can be heavily dependent on the distribution of (pathological) features in the training data. When presenting such a model with imaging that entails new characteristics, which were underrepresented or not included in the training data, model output can be unreliable [45]. This issue was encountered during the evaluation process of this study. Specifically, in a minor subset of cases, with patients who previously underwent decompressive craniectomy. Model 1 was unable to adequately represent this in its output sCTs. The model was, however, able to recognize that there is an irregularity compared to radiographs without craniectomy (Fig. 4E). Similarly, all patients included in the training of model 1 suffered from hydrocephalus. Thus, this model will likely not be as reliable for patients with different diagnoses. In a worst-case scenario, this could lead to the misdiagnosis of patients. Nonetheless, correct model application on images that are representative of the data the model was originally trained on can manage this issue. Currently, it is unclear whether a model like the one we propose can be utilized for diagnostic purposes. It is essential that a separate clinical trial is conducted for each distinct clinical question or diagnostic application to address that question. Images from these craniectomy patients were still kept in the internal validation dataset and, therefore, included in the final performance analysis to obtain a more realistic evaluation of our models. It is essential that future efforts in this area include an assessment of model performance stratified according to pathologies like intracranial bleeds or calvarial fractures, as well as neurosurgical devices like deep-brain stimulation electrodes or ventriculoperitoneal shunts.

Another issue was encountered during the external validation of model 1 and training of model 2: the CT scans used in this study rely on indirect reconstructions for sagittal and coronal views from the axial view, which was the only plane available in high resolution. This posed problems in the Bologna and South Korea datasets, as the number of axial image slices was much lower (roughly 40-80) compared to the Zurich dataset, which generally included CTs with over 200 axial slices. Consequently, the sagittal and coronal reconstructions had low resolution and introduced reconstruction artifacts (see Fig. 5).

For model 1, this impacted the quality of the resulting DRRs in the external validation and, consequently, also affected model performance at external validation, as it introduced a systemic difference compared to the training data. Since validation with an external dataset is essential for establishing generalizability, these data were still included in the results regardless of their suboptimal resolution.

A similar issue was encountered during the training of model 2: As the gtCTs used in training were of low resolution for coronal and sagittal planes, the resulting model-generated CTs also lacked clarity in these planes.

Training of a 2D-to-3D model is immensely computationally expensive. The machine used for training model 1 was equipped with two NVIDIA RTX A6000 GPUs, one of NVIDIA’s best-performing GPUs currently on the market. Still, computational constraints required us to downsample both X-rays and CTs. All training had to be performed locally within the premises of the hospital to fulfill the criteria set by the local ethics committees. The current output resolution of the DRR model is 192 × 192 × 192 voxels, less than half of the standard in-plane resolution of a regular cranial CT. This compromise in resolution naturally means that subtle anatomy might not be represented in sufficient detail. However, early CT images had a similarly low resolution, and only over time were the high-resolution scanners we use today developed [46]. The resolution of the sCT generated as a part of this study was sufficient to assess larger anatomical structures, like skull diameter and ventricle width, however, a more subtle analysis of anatomical structures is outside the realm of this feasibility study. With advances in developing newer and more powerful hardware at affordable prices, it is realistic that a model like the one we present can soon be trained for sCT generation closer to native resolution. As previously touched upon, it is unrealistic to fully match native CT resolution based on the limited information provided by X-rays. Rather, X-ray-to-CT reconstruction models could find their place in detecting broader pathological processes (e.g., significant bleeding or changes in ventricle width) or aiding with intraoperative orientation navigation (e.g., EVD placement), where spatial fidelity is of higher importance than high resolution.

Finally, it is important to acknowledge the preliminary nature of this study, and as such, additional efforts are needed for model enhancement (e.g., improved pre-processing, more high-quality training data, and external validation of model 2). Generating high-quality sCTs from real X-rays poses some major challenges, one of them being the necessity for highly standardized input parameters such as recording angle and image dimensions. The sCT generated by model 2 was of significantly lower quality than those of model 1. While this issue has been partially addressed in this study by co-registering all X-rays to a standardized radiograph, our rigid transform algorithm does not work perfectly, and future studies should aim to improve on this by further optimizing the standardization process at the stage of X-ray acquisition and pre-processing. Moreover, they should include a qualitative assessment by specialists (such as neuroradiologists or neurosurgeons) to compare the results of the proposed reconstruction method against the ground-truth CTs.

Based on imaging of over 1500 CT scans, two GANs for the creation of synthetic cranial computed tomography scans from biplanar radiographs were trained and evaluated, one using DRRs and the other genuine X-rays as input. Even though the sCT scans produced by both models fall short of replicating the accuracy of real CT scans, this proof-of-concept study is the first of its kind to explore this approach for cranial imaging. It provides an early insight into the potential applications of cranial 2D-to-3D medical image conversion.

## Supplementary information

ELECTRONIC SUPPLEMENTARY MATERIAL

## Data Availability

The material in support of our findings can be obtained upon reasonable request from the corresponding author. No imaging data can be shared to protect patient privacy.

## Abbreviations

ASSD: Average symmetric surface distance
COM: Center of mass
DRRs: Digitally reconstructed radiographs
GANs: Generative adversarial networks
gtCT: Ground truth CT
PA: Principal axis
SSIM: Structural similarity index measure
sCT: Synthetic CT
