# Supplementary material for: TomoRay cranial: synthesis of cranial CT imaging from biplanar radiographs using a generative adversarial network
Source: Eur Radiol. 2026 Jan 15;36(6):4873–87. doi: 10.1007/s00330-025-12253-1 (PMC13212724; doi:10.1007/s00330-025-12253-1)
Supplement: Supplementary file 1 — ELECTRONIC SUPPLEMENTARY MATERIAL [file 330_2025_12253_MOESM1_ESM.pdf]

TomoRay Cranial: Synthesis of Cranial CT Imaging from Biplanar Radiographs using a Generative Adversarial Network

ELECTRONIC SUPPLEMENTARY MATERIAL

Supplementary Table S1: Performance of rigid 3D coregistration of Zurich and Bologna datasets.

n = 164	**Before**	**After**	**Improvement**
**DICE Score**			
mean ± SD	0.828 ± 0.043	0.842 ± 0.044	+0.014 (+1.65%)
median (IQR)	0.831 (0.801 - 0.860)	0.851 (0.809 - 0.873)	+0.019 (+2.33%)
**Average Symmetric Surface Distance (ASSD) [mm]**			
mean ± SD	12.667 ± 2.844	11.616 ± 3.054	-1.051 (+8.30%)
median (IQR)	12.901 (11.019 - 14.717)	12.272 (9.453 - 13.864)	-0.628 (+4.87%)
**Distance between centers of masses (ΔCOM) [mm]**			
mean ± SD	11.793 ± 4.446	8.816 ± 3.869	-2.977 (+25.24%)
median (IQR)	11.140 (9.068 - 13.934)	9.227 (5.811 - 11.448)	-1.912 (+17.17%)
**Principal Axis (PA) angle [degrees]***			
mean ± SD	19.307 ± 13.858	18.171 ± 12.870	-1.136 +5.88%
median (IQR)	15.231 (9.639 - 25.586)	14.980 (9.265 - 23.642)	-0.251 +1.65%

SD: standard deviation; IQR: interquartile range

*PA is relatively error-prone for cranial CTs and should therefore be treated as a qualitative metric rather than a precise qualitative assessment of rotational error.

Supplementary Figure S1: Three examples of less successful reconstructions by model 1 on the external validation dataset.

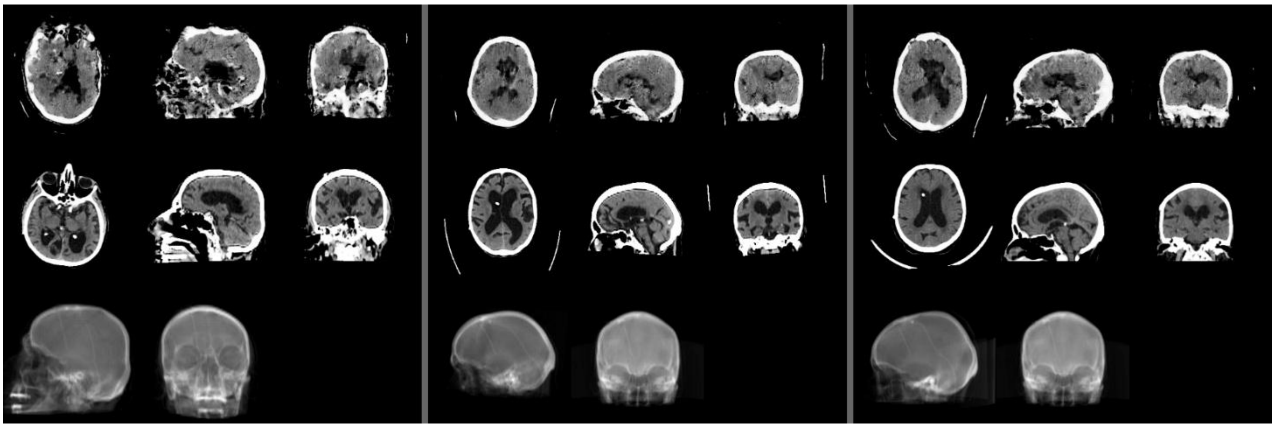

First row: synthetic CTs generated by the GAN

Middle row: corresponding ground truth CT

Last row: digitally reconstructed radiographs used as model input

No corresponding X-rays were available for the CT scans in the external validation dataset.

Supplementary Figure S2: Illustration of rigid coregistration and DICE Score calculation for a case taken from model 1's internal validation dataset.

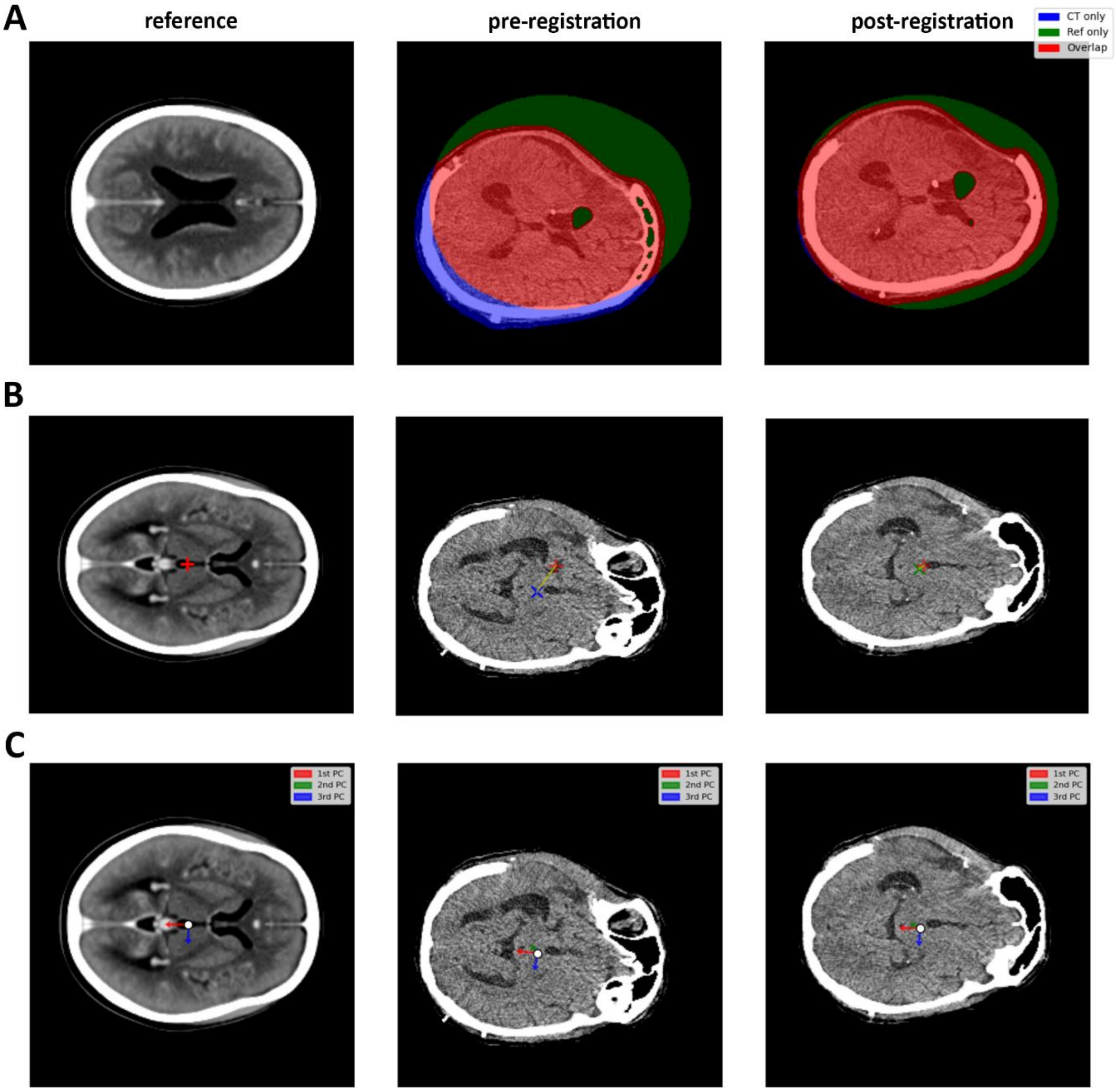

A) DICE Score
B) Distance between Centers of masses (COM distance)
C) Principal axes. Only the most dominant PC1 was used for analysis.
Registration was carried out on full Hounsfield unit range CTs. Windowed CTs are only provided for illustration purposes in this Figure. All measures were calculated on the full 3D volumes.

Supplementary Figure S3: Illustration of automated Cephalic index (CI) calculation from skull masks for model 1

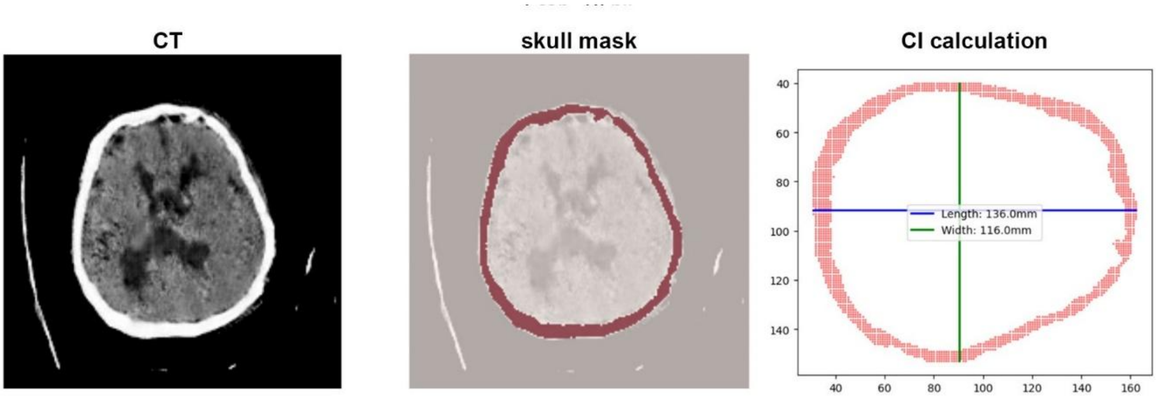

Supplementary Figure S4: Illustration of the automated assessment of skull overlap between real and synthetic CT for model 1.

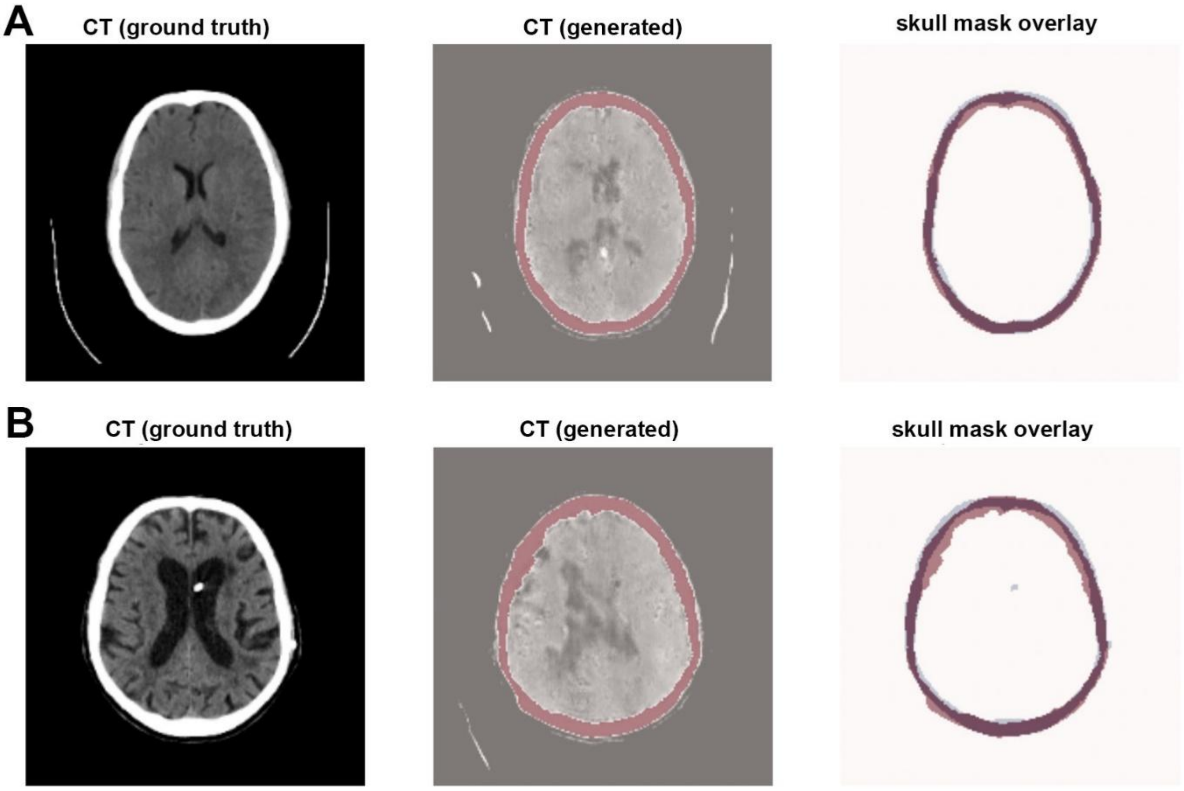

A: Example from internal validation set.
B: Example from external validation set.

Internal validation dataset (n = 34):
Mean Dice: 0.683 ± 0.069
Median Dice: 0.699 (0.657 – 0.722)

External validation dataset (n = 50):
Mean DICE: 0.594 ± 0.096
Median DICE: 0.622 (0.558 - 0.661)

Supplementary Figure S5: Correlation of clinical image features between original and synthetic CTs for the internal (blue) and external (red) cohort used in the assessment of model 1.

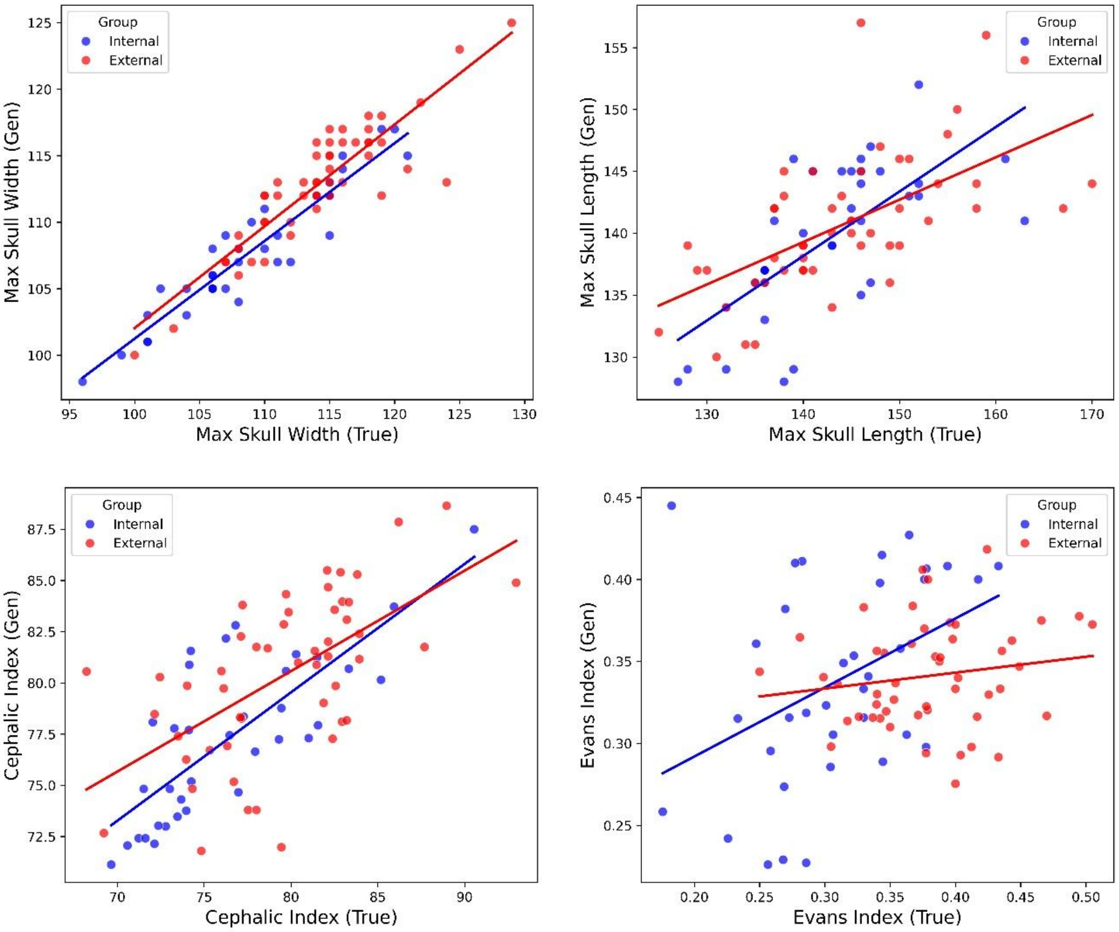

Pearson Correlation Coefficients
Max skull width: internal r = 0.94, external r = 0.89
Max skull length: internal r = 0.69, external r = 0.60
Cephalic index: internal r = 0.78, external r = 0.60
Evans index (manual): internal r = 0.41, external r = 0.16
